# Risk factors for extremely serious road accidents: Results from national Road Accident Statistical Annual Report of China

**DOI:** 10.1371/journal.pone.0201587

**Published:** 2018-08-01

**Authors:** Guodong Liu, Siyu Chen, Ziqian Zeng, Huijie Cui, Yanfei Fang, Dongqing Gu, Zhiyong Yin, Zhengguo Wang

**Affiliations:** 1 The Eighth Department, State Key Laboratory of Trauma, Burn and Combined Injuries, Research Institute of Surgery, Daping Hospital, Army Medical University, Chongqing, P.R. China; 2 Department of Epidemiology and Biostatistics, Southwest School of Medicine and First Affiliated Hospital, Army Medical University, Chongqing, P.R. China; 3 The Fourth Department, State Key Laboratory of Trauma, Burn and Combined Injuries, Institute for Traffic Medicine, Research Institute of Surgery, Daping Hospital, Army Medical University, Chongqing, P.R. China; Charite Medical University Berlin, GERMANY

## Abstract

**Background:**

In the past decades, extremely serious road accidents with a death toll over ten in each have become a severe public health problem in China. This study investigates risk factors contributing to extremely serious road accidents, which will be crucial for accident prevention.

**Methods:**

Collecting data from *The Road Accident Statistical Annual Report* openly issued by China’s Traffic Management Bureau of the Public Security Ministry for the time period 2004–2015, we used the monthly case number of extreme serious road accidents as the dependent variable. We then selected ten risk factors as primary independent variables: professional driver, driving under influence (alcohol or drug), fatigue, vehicle type, overload, brake problem, weather, road classification, terrain, and region. The method of negative binominal regression was implemented to investigate the association between these risk factors and extremely serious road accidents.

**Results:**

A total of 346 extremely serious road accidents were included in our analysis. On a national scale, we found that professional driver [incidence rate ratio (IRR): 1.10, 95% CI: 1.02–1.19], fatigue (IRR: 1.15, 95% CI: 1.03–1.29), large vehicle type (IRR: 1.11, 95% CI: 1.03–1.21), overload (IRR: 1.09, 95% CI: 1.03–1.16), and terrain (IRR: 1.09, 95% CI: 1.01–1.18) were significantly associated with extremely serious road accidents. Besides, separate analyses on western and non-western region indicated that both regions had shared risk factors as well as distinct factors.

**Conclusions:**

Our study identifies professional driver, fatigue, large vehicle type, overload, and terrain as significant risk factors of extremely serious road accidents in China, and targeted and preventative measures could be taken based on our findings.

## Introduction

The World Health Organization reported a global estimate of 1.25 million road traffic deaths per year, and one fifth of the deaths occurred in China [1]. Automobile accidents resulted in 3.6 out of 18.8 deaths per 100,000 people in China in 2013 [1]. However, the vehicle parc in the US was 4.2 times of that in China in 2014 while the number of road traffic deaths in the US was merely 1.9 times of that in China in 2013 [2–3]. Therefore, the road accident death is a public health issue in China and requires comprehensive investigation. Among the road accidents, special attention should be paid to the extremely serious road accidents (ESRAs). Based on the Decree of the State Council, ESRA refers to a traffic accident that causes more than 10 deaths, more than 50 serious injuries, or more than 50 million Yuan (approximately 7.9 million dollars) of direct economic losses [4]. According to *The Road Accident Statistical Annual Report* issued by China Traffic Management Bureau of the Public Security Ministry, 925 ESRAs occurred from 1993 to 2012 with an annual average of 46 cases [5]. In spite of a relatively lower incidence than general road accidents, ESRAs cause substantial losses. Due to the limited samples and incomplete case records, the researches on ESRAs are rare and still at the preliminary stage. Few reports on ESRAs can be found from other countries besides China through Pubmed and Google Scholar. Currently, we could only retrieve one conference report and three degree theses through exhaustive search in the database of Chinese National Knowledge Infrastructure by the relevant terms of extremely serious road accidents [6–9]. The conference report revealed considerable risk factors such as overload by analyzing the ratio of each risk factor per year (i.e., the count of accidents involving a specific factor in one year / the total count of accidents this year) [6]. Nonetheless, the report could not convincingly support the association between factors and ESRA outcome. One thesis on ESRAs found that trucks, improper operation, speeding, abnormal weather, and advanced highway were significant risk factors [7]. However, this study has not involved several important factors such as regions, fatigue driving, driving under influence, etc. Another study revealed that abnormal weather, speeding, and overload were the major inducements of ESRAs by principle component analysis, association rule mining technique, and fault tree model in 2016 with a small sample size of only 139 cases [8]. XL Du’s thesis focused on the driver-related factors, however not considering the category whether driver was professional or not [9]. Overall, the complexity and uncertainty of ESRAs baffled the exploration of causal relationship which dented its prevention. There is an incontrovertible need to explore the risk factors and understand ESRAs comprehensively based on a large sample.

In this study, based on previous literature about road traffic accidents, we included factors of four categories: human factor, vehicle factor, road factor, and environmental factor [10–11]. Among environmental factors, geographic factor should be taken into special consideration in China because of the huge disparities between western and non-western region. There are evident differences between the western region and non-western region in the aspects of terrain, climate, population, and economics. According to the geographical position and economic development, the 32 provinces or cities of China are divided into western region and non-western region (middle and eastern regions). The terrain of western region is mainly plateaus, mountains, and basins while that of the middle and eastern region is plains and hills [12]. These differences may have impacts on identifying risk factors of ESRAs, so the comparison of factors distribution and their effects between western and non-western region is necessary.

By reviewing the records of ESRAs from 2004 to 2015, our study aims to accomplish a comprehensive risk factor profile of ESRAs in China and provide references for further prevention.

## Methods

### Study design

This study was a retrospective observational study using data from *The Road Accident Statistical Annual Report* openly issued by China Traffic Management Bureau of the Public Security Ministry for the time period 2004–2015 [5].

### Data collection, quality control, and confidentiality of privacy

Raw data were collected by the traffic police on-spot, including driver’s characteristics, vehicle status, road conditions, environmental factors, crash time, casualty and injuries, initial travel time, rest time, trace marks after braking, and driver’s blood alcohol concentration. The sample of vehicle wreckage was also sent to professional fault assessment institution. The raw data and identification reports were evaluated by experts from three national institutes, namely, Chinese Ministry of Public Security, Ministry of Transport, and State Administration of Work Safety (S1 File). The assessment results were refined in the reports by Traffic Management Bureau of the Public Security Ministry. The researchers in our study did not contact with the participants directly and had no access to the information that could identify individual participants.

### Variable definition, variable classification, and data sources

The independent variable was the monthly case count of a specific factor. We sorted the factors into four categories: human factor, vehicle factor, road factor, and environmental factor. Human factors are defined as the factors restricting the capability of driving or promote risk-taking behaviors on a short-term or long-term basis, such as aging, profession, fatigue, speeding as a habit, moderate ethanol intake, etc. [10, 13–14]. Vehicle factors include all the factors related to vehicle’s safety status, such as vehicle age, vehicle type, overload condition, insurance condition, and whether the vehicle served for commercial purpose [11, 15]. Road factor refers to the classification of road grades and so on [11, 15]. Environmental factors refer to the influence of time and space, such as the month and year of occurrence, the time point of a day, the accident on weekend or not, the accident in public holiday or not, light condition, visibility, and weather [11, 15]. The classification of factors, data collection, and criteria/judgment resource of variables were shown in Table 1. The count assignment of each factor was shown in S1 Table.

**Table 1:** Independent variable classification, data collection, and criteria.

Variables	Data collection	Criteria/Judgment
**Human factors**		
Professional driver	Employment record and interview of driver collected by the police	Employment status whether the driver takes driving as a profession
Driving under influence (alcohol or drug)	Blood sample of driver	Threshold and test of blood and breath alcohol content of vehicle drivers (GB/T1952) [16]
Fatigue	Starting travel time and rest time during the journey, trace marks of vehicle after braking	Regulations for the implementation of road traffic safety law [17]
Driving without license	Driving license	
Illegally carrying passengers	Passenger limit on license, passenger number	
**Vehicle factors**		
Vehicle type	Type on vehicle license, photos taken by police on the scene	
Overload	Weight/passenger limit on license, passenger number, weight record through transport company and toll station	
Brake problem	Vehicle wreckage	Fault assessment institution
**Environment and road factors**		
Weather	Weather record of Meteorological Bureau, real time record on the scene	
Road classification	Road category record in Urban and rural construction commission	
Terrain	Road curved degree record in Urban and rural construction commission, measurement of the scene	
Region	Record of the provinces	Criteria from National Bureau of Statistics

### Variables selection and statistical analysis

Statistical analysis was done using Stata (Version 11.0, Stata Corp., College Station, TX, USA) and R (Version3.4.1). We conducted Spearman’s correlation to select independent variables. Based on the Spearman’s coefficients and experience, we excluded the factors of illegal carrying passengers and driving without license to reduce overlapping effects. At last, ten factors including professional driver, fatigue, driving under influence, vehicle type, overload, brake problem, road classification, terrain, region, and weather were entered as independent variables.

A preliminary review of scatter plots was performed which indicated that the potential association may not be linear. Because the outcome variable was a count (the number of ESRAs each month), we deliberated on the options of Poisson regression model, zero-inflated regression model, and negative binomial regression model. Due to the over-dispersion, the negative binomial regression model without zero-inflation adjustment was selected. Univariate negative binomial regression analysis was done first. Then, negative binomial regression analysis with ten variables was conducted to explore the factors associated with increased risks of ESRAs in China. After that, further analysis was developed using the subgroup data which was arranged according to the different regions where the ESRAs occurred. The variance inflation factors (VIFs) among independent variables were calculated in order to diagnose the multicollinearity.

Afterwards, in this study, seasonal and long term patterns in both exposure and outcome data could dominate the crude association which usually made the short-term associations of interest hard to detect. In order to explore the relationship between the exposures and short-term variation around long-term patterns, there was a need to control for long-term patterns explicitly. Therefore, a flexible spline function of time was applied.

## Results

### Descriptive statistics

**Fig 1:**
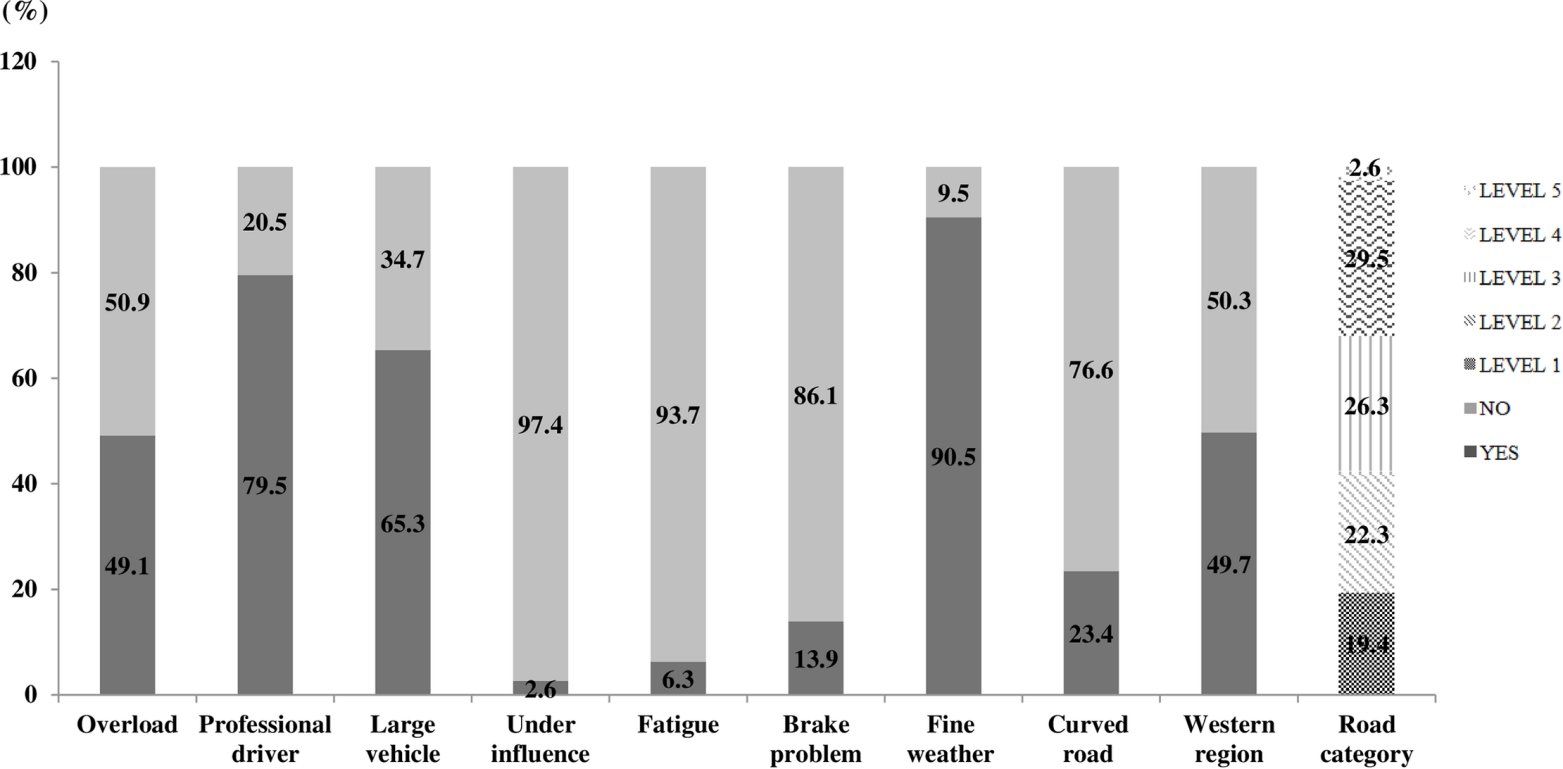
Distribution of ESRA by human, vehicle, road, and environmental factors.

A total of 346 ESRAs were reported nationally from 2004–2015, with an annual average of 29 cases. For human factors, the numbers of professional drivers, driving under influence (alcohol or drug), and fatigue driving were 275 (79.5%), 6 (1.7%), and 22 (6.4%), respectively. For vehicle factors, the cases with overload condition and brake problem were 170 (49.1%) and 48 (13.8%). A total of 65.3% vehicles were in large size, and only 13.3% were in small size. For road and environmental factors, 33 cases (9.5%) occurred in abnormal weather, and the number of curved road was 81 (23.4%). Western region occupied a huge proportion of ESRAs (49.7%) with 172 cases compared with middle and eastern regions. Specifically, three provinces in southwestern region of China (Yunnan, Sichuan, and Guizhou) were the three leading provinces of ESRA number (32, 25, and 22 respectively). These data with more details were shown in Fig 1.

### Factors associated with nationwide ESRAs

Univariate analysis using negative binomial regression model indicated statistically significant associations between ESRAs and professional driver (driver who takes driving as profession), fatigue, driving under influence, vehicle type (large vehicle type), overload, brake problem, terrain with curve, weather (abnormal weather), road classification (advanced road), and region, respectively. According to the results of best fitting multivariate model, as shown in Table 2, compared with the group of unprofessional drivers, there was a higher risk in professional driver group [incidence rate ratio (IRR): 1.10, 95% CI: 1.02–1.19)]. Further, a higher risk was found in fatigue group (IRR: 1.15, 95% CI: 1.03–1.29). In the aspect of vehicle factors, large vehicle type was significantly associated with an increase of 11% in ESRA count (IRR: 1.11, 95% CI: 1.03–1.21), and overload accounted for an increase of 9% in ESRA count (IRR: 1.09, 95% CI: 1.03–1.16). Monthly ESRA count was weakly associated with terrain with curve (IRR: 1.09, 95% CI: 1.01–1.18). There was no evidence suggesting a relationship between ESRAs and driving under influence, brake problem, advanced road, weather, and region in the final model. The VIFs varied from the lowest 1.18 (driving under influence) to the highest 9.56 (professional driver), all of which were less than ten.

**Table 2:** Negative binomial regression coefficients of nationwide analysis.

Variables	Std.Error	Z value	P value	IRR	95%Confidence Interval	
Professional driver	0.04	2.54	0.011	1.10	1.02	1.19
Fatigue	0.06	2.39	0.017	1.15	1.03	1.29
Vehicle type^a^	0.04	2.57	0.010	1.11	1.03	1.21
Overload	0.03	2.96	0.003	1.09	1.03	1.16
Terrain	0.04	2.33	0.020	1.09	1.01	1.18
DUI^b^	0.13	0.92	0.358	1.13	0.87	1.45
Brake problem	0.05	1.45	0.146	1.07	0.98	1.18
Road classification ^c^	0.04	1.59	0.113	1.06	0.99	1.15
Weather	0.06	1.25	0.211	1.08	0.96	1.21
Region	0.03	1.73	0.084	1.05	0.99	1.12

^a^Vehicle type represents the type of large vehicle.

^b^DUI stands for driving under influence.

^c^Road classification is the factor of advanced road.

### Factors associated with western and non-western ESRAs

**Fig 2:**
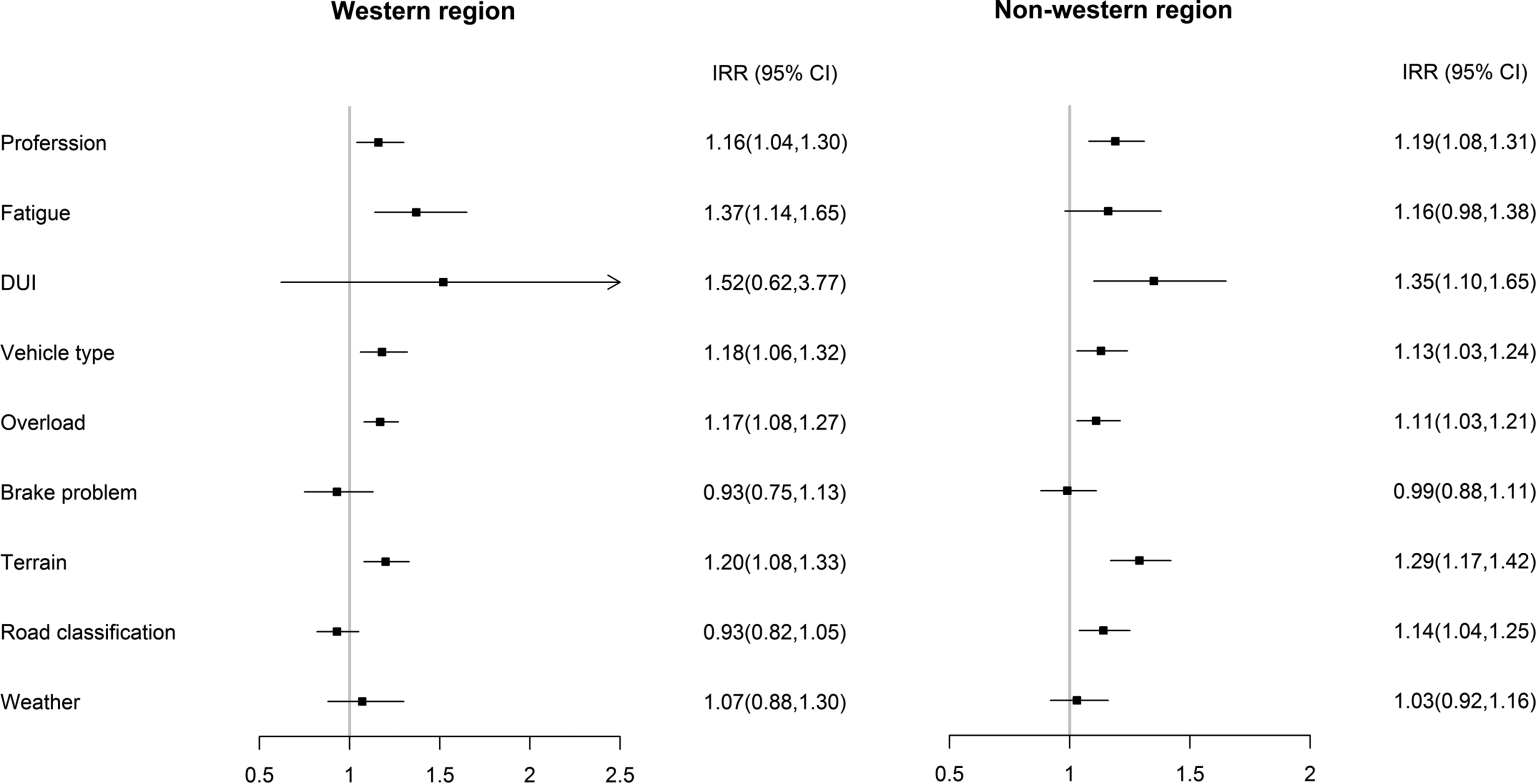
The negative binomial regression coefficients in two models of western and non-western regions.

The subgroup analysis of western and non-western regions showed both similarities and differences. Fig 2 showed that in China’s western region, increased risks were also found in group of professional drivers (IRR: 1.16, 95% CI: 1.04–1.30) and drivers with fatigue (IRR: 1.37, 95% CI: 1.14–1.65). For the vehicle factors, the overload condition (IRR: 1.17, 95% CI: 1.08–1.27) and large vehicle type (IRR: 1.18, 95% CI: 1.06–1.32) showed higher risks. The terrain with curve (IRR: 1.20, 95% CI: 1.08–1.33) was significantly and positively associated with the number of ESRAs. For ESRAs in non-western region, the number increase was associated with professional driver, driving under influence, vehicle type, overload, terrain, and road classification. The professional driver was significantly associated with an increase of 19% in ESRA count (IRR: 1.19, 95% CI: 1.08–1.31) and another human factor driving under influence was significantly associated with an ascending of 35% in ESRA monthly sum (IRR: 1.35, 95% CI: 1.10–1.65). Large vehicle type (IRR: 1.13, 95% CI: 1.03–1.24) and overload (IRR: 1.11, 95% CI: 1.03–1.21) were significantly and positively associated with the count of ESRAs. An elevated risk was also identified in the road and environmental factors of terrain with curve (IRR: 1.29, 95% CI: 1.17–1.42) and advanced road (IRR: 1.14, 95% CI: 1.04–1.25).

## Discussion

Using data of 346 ESRAs from *The Road Accident Statistical Annual Report* openly issued by China’s Traffic Management Bureau, we analyzed ten risk factors and identified professional driver, fatigue, large vehicle type, overload, and terrain as the significant risk factors of ESRAs on a national scale. Besides, separate analyses on western and non-western regions revealed that these two regions had shared risk factors as well as distinct factors.

### The factors associated with ESRAs

Our analysis indicated that professional driver was a risk factor associated with ESRAs, consistent with previous studies. A study showed professional drivers had more than half accident rate than ordinary drivers did without adjustment of professional drivers’ higher mileage [10]. McKenna suggested occupational stress of company car drivers as an essential predictor of accident rates [18]. Consistently, in our study, 79.5% drivers were professional drivers, and thus the professional driver might be responsible for the increase. The factor of professional driver could combine with other factors such as driver’s gender, mobile phone using, lifestyle, and poor responsibilities [19–21].

Another factor fatigue also showed significant association with ESRAs. Fatigue was defined as driving for more than four hours without at least 20 minute rest in China [17]. In United States and United Kingdom, sleepiness and fatigue were the causes of traffic fatalities in 2.6% and 3% of all cases, respectively [3]. Some previous studies reported that a complaint of excessive daytime sleepiness, which could have similar effects of fatigue, was positively associated with significantly increased traffic accident rate in commercial truck drivers during long-haul [22–24]. Moreover, the scholars identified male drivers, trucks, driving during midnight to dawn, and morning rush hours as risk factors of fatigue-related crashes [25].

Large vehicle type exerted significant influence on the risk of ESRAs. Greek researchers have reported that compared with car (8.4%) and bus (3.6%), the ratio of death versus all the people involved was as high as 12.7% in truck accident [26]. In United States, a total of 3757 people were killed in accidents involving large trucks in 2011 and the large truck accident related fatalities increased 11% from 2009 to 2011 [27]. In our study, large vehicles occupied a substantial proportion (65.32%). Other lines of evidence showed that the judgments of drivers would be affected by head-on large vehicles on the road [28]. Therefore, large vehicle type is a significant risk factor.

Overload was found to be a risk factor of ESRAs in our study. Previous literature indicated higher speed was observed in heavier cars [29]. Overload was correlated with other traffic violations and presented an increased risk of severe accidents [11]. Among the speeding cases, the overload proportion was as high as 11.66% [30]. The association between overload and other violations in ESRAs could be explored in the future. In other countries, overload reports were scant. Overload accounted for 5.1% in Kenya’s traffic crashes in 1990 [31]. The inadequate transport payment in Colombia according to the number of passengers caused drivers to choose overload for more profits [32]. In the year of 2001–2003 in China, based on the examination of four provinces, the ratio of overload transport vehicle was over 40% [33]. And the overload condition accounted for a substantial proportion of 49.13% in ESRAs. The transport capacity surpassing demand lowered the price and caused the drivers to choose overload to earn more money, which could be a vicious circle.

The terrain with curve was found to be a significant factor in nationwide analysis of ESRAs. Regarding to the terrain, previous studies observed the strong association between adverse geometric terrain and location with high accident rate [34]. In the survey conducted by Chinese government, the fatality rate in curved road was as high as 0.8, while the average fatality rate was 0.3 among all crashes in 2014 [35]. The finding that higher accident rate was associated with smaller radius of curvature corroborated the effect of curved pathway on ESRAs [36]. In general, accident rate increased consistently with the increased degree of horizontal curvature [37].

### Other potential factors

Region was not a significant factor in the nationwide analysis of ESRAs. However, the western region has half gross regional product of eastern region’s (12038 billion vs. 45075 billion) and population of western region was less than one third of non-western region [38–39]. Weather and climate condition incurred not only the instant effect of precipitation for traffic crashes, but also potential effects of certain economic and social variables [40]. The western region has all kinds of terrains while the non-western region has mainly plains and hills, which influence the weather and climate patterns [12]. The area covering Sichuan, Yunnan, Guizhou, and Chongqing in the western region had the highest ESRA incidence. The common significant risk factors of two regions were professional driver, vehicle type, overload, and terrain. The unique significant risk factor of western region was fatigue while the particular significant risk factors of non-western regions were driving under influence and advanced road.

Driving under influence, accounting for 1.7% of ESRAs, was a significant factor in non-western region subgroup analysis. In United States, Japan, Germany, and Spain, drunk driving accounted for 31%, 7.2%,7.8%, and 25% in all fatal crashes in 2014, respectively [3, 41]. Obviously, driving under alcohol/drug influence was considered to be a factor of traffic accident because of the temporary physiological changes [18].

The advanced way factor indicated in non-western region, driving on high expression way, national road, provincial road, urban road, and high-class road would have more risk of ESRA. In an analysis of high expression way traffic injuries in China, the driving experience year >20 years, absence of license, foggy and dusty day, dry road surface, and the traffic violation behavior of pedestrian/driver were risk factors [42].

Weather is a common factor in traffic accidents, and drivers tend to take cautions in bad weather [43–44]. However, weather factor was not significantly correlated with the number increase of ESRAs. According to the descriptive statistics, most of ESRAs happened in fine weather, which was consistent with an analysis in England and Wales that more than 70% accidents were reported in fine weather without high wind [45].

### Future prevention indication

Our findings could indicate directions for future prevention. For professional drivers, driving safety education should be enhanced on a regular basis. Overload condition and brake maintenance awareness should be paid attention to, especially in large vehicles [46]. The high penalty fee in China might be feeble for the overload inhibition compared with the UK [47–50]. Automated overload detection device and road fee charge based on weight could be implemented at the same time [51–52]. More warning signals for curved road are imperative during the redesign and reconstruction of places with high traffic accident rate.

### Limitations

Our study has several limitations. Firstly, the raw data collected by the police at the site could be biased due to subjective judgment. Secondly, the corresponding hospital records and medical appraisal standard for excess daytime sleepiness were absent. Therefore, data collection process with standardized method should be implemented in the future to produce more comprehensive data. Besides, the socioeconomic factors such as population and gross region product could be explored for better estimation of region disparities. Moreover, general traffic accident data as the control could be included for comparison in the future.

## Conclusion

The overall findings suggest professional driver, fatigue, large vehicle type, overload, and terrain play important roles in the occurrence of ESRAs. And subgroup analysis of region reveals more risk factors such as driving under influence and advanced road in non-western region. Further investigation is needed on the basis of stratification for future prevention.

## Supporting information

S1 File Raw data. Specific information of each case was list in the raw data. The 0 means the case without corresponding condition and the 1 means the case with corresponding condition.
(XLS)

S1 Table The assignment of independent variables. Each factor was divided into different categories. Different categories were assigned in different values in each case. The value of one independent variable was the sum of all the cases’ values in one month.
(DOCX)
