# Supplementary material for: Risk factors for extremely serious road accidents: Results from national Road Accident Statistical Annual Report of China
Source: PLoS One. 2018 Aug 1;13(8):e0201587. doi: 10.1371/journal.pone.0201587 (PMC6070265; doi:10.1371/journal.pone.0201587)
Supplement: S1 Table — Each factor was divided into different categories. Different categories were assigned in different values in each case. The value of one independent variable was the sum of all the cases’ values in one month. (DOCX) [file pone.0201587.s002.docx]

**S1 Table. The assignment of independent variables.**

| **Factors** | **Variables** |  | **Variable assignment** |
| --- | --- | --- | --- |
| Human factors | Whether the driver was professional driver | Yes | 1 |
|  |  | No | 0 |
|  | Driving under influence (alcohol or drug) | Yes | 1 |
|  |  | No | 0 |
|  | Driving with fatigue | Yes | 1 |
|  |  | No | 0 |
|  | Driving without license | Yes | 1 |
|  |  | No | 0 |
|  | Illegally carrying passengers | Yes | 1 |
|  |  | No | 0 |
| Vehicle factors | Vehicle type | Large | 1 |
|  |  | Middle | 0 |
|  |  | Small | 0 |
|  | Overload | Yes | 1 |
|  |  | No | 0 |
|  | Brake problem | Existence | 1 |
|  |  | Absence | 0 |
| Road and environmental factors | Weather | Rainy | 1 |
|  |  | Snowy | 1 |
|  |  | Foggy | 1 |
|  |  | Sunny | 0 |
|  |  | Cloudy | 0 |
|  | Road classification | Expressway | 1 |
|  |  | National road | 1 |
|  |  | Provincial road, urban road,  first-grade highway, and high-class highway | 1 |
|  |  | County road, township road, and village road | 0 |
|  |  | Road under construction, depopulation zone road, self-built road, and scenic spot road | 0 |
|  | Terrain | With curve | 1 |
|  |  | Without curve | 0 |
|  | Region | Western | 1 |
|  |  | Middle | 0 |
|  |  | Eastern | 0 |
